# Heat Stress and Thermal Perception amongst Healthcare Workers during the COVID-19 Pandemic in India and Singapore

**DOI:** 10.3390/ijerph17218100

**Published:** 2020-11-03

**Authors:** Jimmy Lee, Vidhya Venugopal, P K Latha, Sharifah Badriyah Alhadad, Clarence Hong Wei Leow, Nicholas Yong De Goh, Esther Tan, Tord Kjellstrom, Marco Morabito, Jason Kai Wei Lee

**Affiliations:** 1 Ng Teng Fong General Hospital, Singapore 609606, Singapore; gummyberryjuice@msn.com (J.L.); esther_tan_xi_xiang@nuhs.edu.sg (E.T.); 2 Sri Ramachandra Institute of Higher Education and Research, Chennai, Tamil Nadu 600116, India; vvidhya@ehe.org.in (V.V.); latha@ehe.org.in (P.K.L.); 3 AMET University, Chennai, Tamil Nadu 603112, India; 4 NUS Graduate School for Integrative Sciences and Engineering, National University of Singapore, Singapore 119077, Singapore; sharifah.b@u.nus.edu; 5 Department of Physiology, Yong Loo Lin School of Medicine, National University of Singapore, Singapore 117593, Singapore; 6 Saw Swee Hock School of Public Health, National University of Singapore, Singapore 117549, Singapore; 7 Human Potential Translational Research Programme, National University of Singapore, Singapore 119228, Singapore; clarenceleow.95@gmail.com (C.H.W.L.); nicholasgoh@nus.edu.sg (N.Y.D.G.); 8 Health and Environment International Trust, Nelson 7005, New Zealand; kjellstromt@yahoo.com; 9 National Centre for Epidemiology and Population Health, Australian National University, Canberra 2601, Australia; 10 Institute of BioEconomy, National Research Council, 50019 Florence, Italy; marco.morabito@ibe.cnr.it; 11 Centre of Bioclimatology, University of Florence, 50144 Florence, Italy; 12 N.1 Institute for Health, National University of Singapore, Singapore 117456, Singapore; 13 Global Asia Institute, National University of Singapore, Singapore 119076, Singapore

**Keywords:** PPE, climate change, worker protection, KAP survey, mitigation strategies

## Abstract

The need for healthcare workers (HCWs) to wear personal protective equipment (PPE) during the coronavirus disease 2019 (COVID-19) pandemic heightens their risk of thermal stress. We assessed the knowledge, attitudes, and practices of HCWs from India and Singapore regarding PPE usage and heat stress when performing treatment and care activities. One hundred sixty-five HCWs from India (*n* = 110) and Singapore (*n* = 55) participated in a survey. Thirty-seven HCWs from Singapore provided thermal comfort ratings before and after ice slurry ingestion. Differences in responses between India and Singapore HCWs were compared. A *p*-value cut-off of 0.05 depicted statistical significance. Median wet-bulb globe temperature was higher in India (30.2 °C (interquartile range [IQR] 29.1–31.8 °C)) than in Singapore (22.0 °C (IQR 18.8–24.8 °C)) (*p* < 0.001). Respondents from both countries reported thirst (*n* = 144, 87%), excessive sweating (*n* = 145, 88%), exhaustion (*n* = 128, 78%), and desire to go to comfort zones (*n* = 136, 84%). In Singapore, reports of air-conditioning at worksites (*n* = 34, 62%), dedicated rest area availability (*n* = 55, 100%), and PPE removal during breaks (*n* = 54, 98.2%) were higher than in India (*n* = 27, 25%; *n* = 46, 42%; and *n* = 66, 60%, respectively) (*p* < 0.001). Median thermal comfort rating improved from 2 (IQR 1–2) to 0 (IQR 0–1) after ice slurry ingestion in Singapore (*p* < 0.001). HCWs are cognizant of the effects of heat stress but might not adopt best practices due to various constraints. Thermal stress management is better in Singapore than in India. Ice slurry ingestion is shown to be practical and effective in promoting thermal comfort. Adverse effects of heat stress on productivity and judgment of HCWs warrant further investigation.

## 1. Introduction

On 11 March 2020, the World Health Organization declared a pandemic due to the spread of the coronavirus disease 2019 (COVID-19). The development of the COVID-19 pandemic was rapid but uneven between India and Singapore. From the time it was declared a pandemic until the end of August 2020, the number of cases in India increased from 60 to 3,542,733 and the number of cases in Singapore increased from 166 to 56,717 [1,2]. In India, this represented a rise from less than 0.001% to 0.26% of the population, while in Singapore, this represented a rise from about 0.003% to about 1% of the population. Pandemics are resource-intensive and can place extraordinary pressure on any country’s healthcare system, depending on the burden of the illness [3]. Potential adverse physical and mental effects experienced by frontline healthcare workers (HCWs) may further stress the healthcare system. HCWs face an increasing demand for patient care, managing logistics, and long working hours. This can be exacerbated by the stress imposed on the body due to the need to wear enhanced personal protective equipment (PPE) which is necessary to reduce the risk of disease transmission [4].

PPE worn by HCWs usually includes a gown, gloves, and N95 respirator with face shield or goggles. As a routine precaution for enhanced environmental and personal hygiene, strict compliance to guidelines on the usage of recommended PPE is emphasized when treating patients with respiratory illnesses, even when COVID-19 is not suspected [5]. While PPE offers protection for HCWs against biological hazards, it subjects the body to additional heat stress [6]. The human body generates heat in the range of about 100 W at rest to 500 W or higher when working, depending on the activity [7]. To maintain homeostasis and avoid heat injury and illness, the body must dissipate most of this heat to the environment via sweat evaporation, convection, and conduction. PPE use strains HCWs physiologically by restricting vapor molecule transfer from the body to the environment. This limits evaporation and heat exchange with the environment [8], thereby increasing the risk of overheating and thermal strain [9]. Thermal stress may be further accentuated by hot and humid weather conditions, proximity to other heat sources, and limitations or lack of cooling provisions. With temperatures in June this year soaring up to 42.2 °C and 32.7 °C in India and Singapore, respectively [10,11], the effects of thermal stress can be a concern for HCWs in both countries during the pandemic.

While studies surveying the knowledge, attitudes, and practices (KAP) of HCWs have emerged [6,12,13], none so far has quantified the KAP of HCWs regarding PPE use and heat stress management during this pandemic. For this reason, the primary aim of this study was to assess the KAP of HCWs from India and Singapore on PPE usage and heat stress when performing treatment and care activities (TCAs) during the COVID-19 global pandemic. The secondary objective was to study the potential benefits of ice slurry ingestion. With the pandemic still ongoing worldwide, we envision that results from this study could serve to improve the understanding of thermal strain experienced by HCWs performing TCAs using PPE and to determine appropriate measures to mitigate heat stress.

## 2. Materials and Methods

### 2.1. Participants

The target population of the questionnaire was HCWs performing TCAs during the COVID-19 pandemic from both India and Singapore. In India, the participants were from institutions from Tamilnadu, Pondicherry, and Andhra Pradesh, which are located in the southern region of India. The ten institutions involved were Apollo Hospital, Narayana Medical College, Nellore; Primary Health Centre, Srikakulam and Government Medical College, Visakhapatnam in Andhra Pradesh; Christian Medical College, Vellore; Kilpauk Medical College, Apollo Hospital and Stanley Medical College in Chennai; and Government Medical College, Thanjavur and Government Medical College, Thiruvarur in Tamilnadu. In Singapore, the participants were from Ng Teng Fong General Hospital Emergency Department, part of the National University Health System. Data collection was conducted in May and June 2020 in hospitals located in Southern India and at Ng Teng Fong General Hospital (NTFGH) Emergency Department in Singapore.

In India, the first COVID-19 cases were reported in March 2020. Air-conditioners were banned in the emergency department and patient care or testing areas, where most HCWs work. A fever clinic was set up outside each hospital with a tent for preliminary consultation and COVID-19 screening. Different types of HCWs wore different levels of PPE. HCWs were advised to doff their PPE during breaks or meals and don a new set afterward.

In Singapore, pandemic operational plans were activated on 7 February 2020. The NTFGH Emergency Department was divided into a “clean operations zone” and a “dirty contamination zone”. In the “dirty zone”, which included a fever facility (FF), liquid impermeable gowns, gloves, goggles, surgical caps, and masks were required. Outside the FF, a fever tentage was also erected to meet the increasing patient load. HCWs working in the tentage were exposed to high heat stress due to long shifts and PPE use in hot weather conditions and poor ventilation.

### 2.2. Design

This study adopted a cross-sectional observational design. A self-designed questionnaire was administered to HCWs performing TCAs to understand perceptions about thermal stress induced by PPE use during the COVID-19 pandemic. On top of the cross-sectional observational study design, an additional experimental design was also employed in Singapore. This is where ice slurry was provided to HCWs so as to determine its effect on thermal stress perceptions. Participation was voluntary and anonymous. In India, ethics approval was granted by Sri Ramachandra Institution of Higher Education and Research (Reference No. IEC-NI/17/APR/59/54). In Singapore, ethics approval was granted by the National Healthcare Group Domain Specific Review Board (Reference No. 2020/00590).

### 2.3. Procedure

#### 2.3.1. Questionnaire

The questionnaire consisted of five sections. The first section gathered HCWs’ demographic data and relevant work information. The second section contained questions on PPE usage at work and other heat-exposure-related questions. The third section examined participants’ adaptation to heat stress and behavioral changes while working in PPE. The fourth section investigated HCWs’ knowledge about thermal stress. The fifth section surveyed HCWs’ attitudes towards PPE use. In the fourth and fifth sections, a 5-point Likert scale was used: 1 for strongly disagree and 5 for strongly agree.

Questionnaires were standardized in English in both countries. In India, hard-copy questionnaires were administered during breaks wherever possible. In less-accessible hospitals, electronic copies were provided. In Singapore, only hard-copy questionnaires were provided either during breaks or towards the end of shifts. In both countries, participation was voluntary and participant informed consent was obtained before administering the questionnaire.

#### 2.3.2. Assessment of Environmental Conditions

In India, ambient temperature, relative humidity (RH), and dew point were recorded using data loggers (EL-USB-2-LCD+, Lascar Electronics, Salisbury, UK). Loggers were placed in selected work areas, about 1.5 m from the ground and walls and 1.5 m from direct heat sources. Wet-bulb globe temperature (WBGT) was estimated using the online heat stress index calculator available in the Climate CHIP tool [14]. In Singapore, ambient dry bulb temperature, wet bulb temperature, globe temperature, WBGT, and RH were measured by a heat stress monitor (QUESTemp QT-44, 3 M, Shoreview, Minnesota, U.S.). Heat stress monitors were placed in the clean zone, the FF, and the tentage. Recordings were made every 5 min for the period monitored. Half-hourly and hourly averages were used to derive WBGT (WBGT = 0.7 × wet-bulb temperature + 0.2 × globe temperature + 0.1 × ambient temperature).

#### 2.3.3. Ice Slurry

In Singapore, ice slurry made from a commercially available sports drink (Pocari Sweat, Otsuka Pharmaceutical, Japan; carbohydrate percentage = 6.2%) was provided. Ice slurry was prepared using a commercially available ice slurry machine (IPRO, SPM Drink Systems, Modena, Italy). Respondents reported their thermal comfort rating on a 7-point scale from cold (−3) to hot (+3) [15] before and after ice slurry ingestion.

### 2.4. Statistical Analysis

The frequency and descriptive statistics of the questionnaire results were compiled and recorded on Microsoft Excel and exported to R version 3.6.2 for analysis. Each continuous variable was tested for normality using the Shapiro–Wilk test. Means with standard deviations (SD) and medians with interquartile ranges (IQRs) were used to describe normally and non-normally distributed variables, respectively. Differences in group medians between Indian and Singaporean respondents were tested using the Wilcoxon rank-sum test for continuous variables, since all of the variables but one (BMI of respondents in India) were found to be non-normally distributed via the Shapiro-Wilk test, and Pearson’s chi-square or Fisher’s exact tests for categorical variables. A *p*-value cut-off of 0.05 represented statistical significance.

## 3. Results

### 3.1. Demographic Details and Work Information

A total of 110 HCWs from India and 55 HCWs from Singapore participated in this study. The median WBGT reported was also higher in India (30.2 °C (IQR 29.1–31.8 °C)) than in Singapore (22.0 °C (IQR 18.8–24.8 °C)) (*p <* 0.001) (Table 1).

The majority of respondents across both countries either held a medical (66 physicians, 40% in total) or a nursing role (61 nurses, 37% in total). Respondents were distributed across a range of work locations in both countries, with a majority being in FFs or tentages in Singapore and no discernible pattern being observed in India (Table 1).

### 3.2. Knowledge on Heat Stress and PPE Use

**Figure 1:**
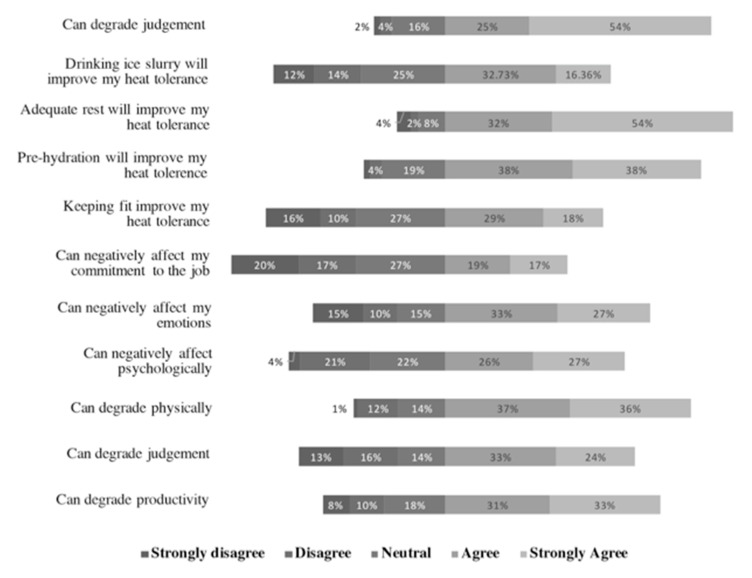
HCW respondents’ responses (total) to statements on knowledge on effects of heat stress on a 5-point Likert scale: (1) strongly disagree; (2) disagree; (3) neither agree nor disagree; (4) agree; (5) strongly agree.

The majority of respondents either agreed (4) or strongly agreed (5) to all statements (on a 5-point Likert scale, “1” for “strongly disagree and “5” for “strongly agree”), except for the following statements: “drinking ice slurry will improve my heat tolerance”, “heat stress can negatively affect commitment to the job”, and “keeping fit can improve heat tolerance” (Figure 1).

Respondents in Singapore agreed more strongly than their counterparts in India with the following statements:

1. “Heat stress can degrade productivity” (Wilcoxon rank-sum test statistics (W) = 2216.5, *p* < 0.01);
2. “Heat stress can degrade judgment” (W = 2207, *p* < 0.01);
3. “Heat stress can negatively affect me psychologically” (W = 1905, *p* < 0.01);
4. “Heat stress can negatively affect emotions” (W = 1817, *p* < 0.01);
5. “Pre-hydration can improve heat tolerance” (W = 3824.5, *p* < 0.01);
6. “Drinking ice slurry will improve heat tolerance” (W = 1898.5, *p* < 0.01).

Respondents in India agreed more strongly with “adequate rest can improve heat tolerance” (W = 3676, *p <* 0.01) than their counterparts in Singapore.

There was no difference in responses to the following statements:

1. “Heat stress can degrade me physically”;
2. “Keeping fit can improve heat tolerance”;
3. “PPE prevents sweat evaporation”.

### 3.3. Attitudes towards PPE Usage

**Figure 2:**
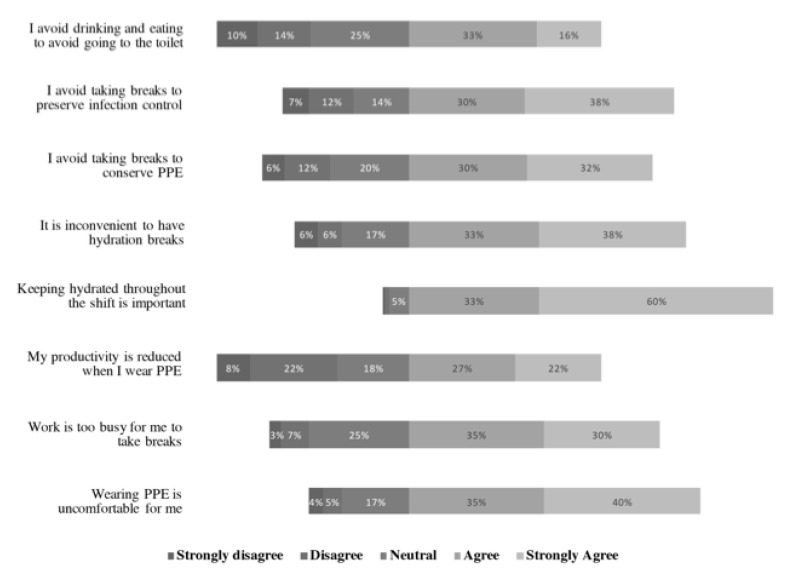
HCW respondents’ responses (total) to statements on attitudes towards personal protective equipment (PPE) usage on a 5-point Likert scale: (1) strongly disagree; (2) disagree; (3) neither agree nor disagree; (4) agree; (5) strongly agree.

Respondents in both countries generally agreed (4) or strongly agreed (5) with all the statements, except “I avoid drinking and eating to avoid going to the toilet” and “my productivity is reduced when wearing PPE” (Figure 2).

More respondents in Singapore than India agreed more strongly that “drinking ice slurry will improve heat tolerance” (W = 1898.5, *p* < 0.01).

Conversely, more respondents in India than Singapore agreed more strongly with the following statements:

1. “Wearing PPE is uncomfortable for me” (W = 3719.5, *p* < 0.01);
2. “I avoid breaks to conserve PPE” (W = 4114.5, *p* < 0.01);
3. “I avoid breaks to preserve infection control” (W = 4492.5, *p* < 0.01);
4. “I avoid eating and drinking to avoid going to the toilet” (W = 3889, *p* < 0.01).

There was no difference in responses to the following statements:

1. “Work is too busy for me to take breaks”;
2. “My productivity is reduced when wearing PPE”;
3. “Keeping hydrated throughout the shift is important”;
4. “It is inconvenient to have hydration breaks”;
5. “PPE will prevent sweat evaporation”.

### 3.4. PPE Usage Practices and Symptoms of Heat Stress

**Figure 3:**
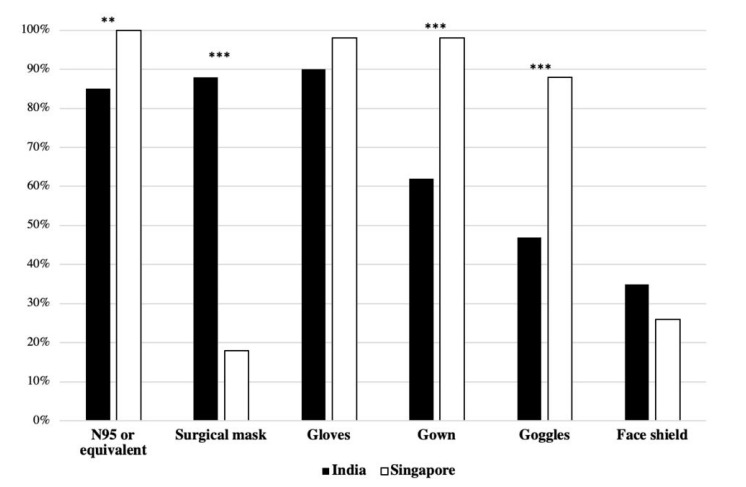
Type of PPE worn by HCW respondents during their shifts in % of respondents in each country (** *p* < 0.01; *** *p* < 0.001).

Various types of PPE were used by HCWs during the pandemic (Figure 3). Use of the following types of PPE was more frequent among HCWs in Singapore than in India.

(1) N95 masks: Singapore (*n* = 55, 100%) vs. India (*n* = 93, 85%) (χ^2^(1) = 7.878, *p* < 0.01);
(2) Gown: Singapore (*n* = 54, 98%) vs. India (*n* = 68, 62%) (χ^2^(1) = 22.93, *p* < 0.01);
(3) Goggles: Singapore (*n* = 48, 88%) vs. India (*n* = 52, 47%) (χ^2^(1) = 22.93, *p* < 0.01).

Use of surgical mask was the only type of PPE more frequently used by HCWs in India (*n* = 97, 88%) than in Singapore (*n* = 10, 18%) (χ^2^(1) = 75.78, *p* < 0.01).

Gloves were worn by majority of HCWs in both India (*n* = 99, 90%) and Singapore (*n* = 53, 98%). Face shields were the least-used PPE in India (*n* = 38, 35%) and in Singapore (*n* = 14, 26%) (Figure 3).

The prevalence of working in an air-conditioned location was also much higher in Singapore (*n* = 34, 62%) than in India (*n* = 27, 25%) (*p* < 0.001). The availability of a dedicated rest area was higher for HCWs in Singapore (*n* = 55, 100%) than in India (*n* = 46, 42%) (*p <* 0.001), and the number of respondents who reported taking off PPE during breaks was also higher in Singapore (*n* = 54, 98.2%) than in India (*n* = 66, 60%) (*p* < 0.001) (Table 2).

Respondents reported experiencing various symptoms associated with thermal stress, except vomiting (Table 2). Symptoms such as thirst (*n* = 144, 87%), excessive sweating (*n* = 145, 88%), exhaustion (*n* = 128, 78%), and wanting to go to comfort zones (*n* = 136, 84%) were highly reported. Only few respondents across both countries (*n* = 7, 4%) took sick leave.

### 3.5. Ice Slurry

Thirty-seven observations of thermal comfort ratings before and after ingestion of ice slurry were recorded amongst the 55 respondents in Singapore. The median rating improved from 2 (IQR 1–2) (warm) before ingestion to 0 (IQR 0–1) (neutral) after ingestion (*p* < 0.001).

## 4. Discussion

The primary objective of this study was to determine the KAP of HCWs from India and Singapore regarding PPE usage and heat stress when performing TCAs during the COVID-19 pandemic. We observed that HCWs wearing PPE while working in hot and humid environments display heat strain symptoms. Our findings suggest that HCWs are aware of the adverse effects of thermal strain and how to mitigate them. However, best practices might not be adopted. The secondary objective of this study was to investigate the potential benefits of ice slurry ingestion. Our study showed an improvement in thermal comfort when HCWs were provided with ice slurry. We postulate that findings from this study can help to improve the understanding of thermal stress levels experienced by HCWs and determine suitable measures to ensure the health and safety of HCW when wearing PPE to perform TCAs.

Based on the questionnaire results, it seems that respondents were aware of the potential adverse physiological, psychological, and mental effects of heat stress. A majority of HCWs also reported that heat stress was able to adversely influence their productivity, judgment, and emotions. This is consistent with other studies that have demonstrated that heat stress reduces work capacity [16], increases errors [17] and accidents [18], and deteriorates health and productivity [19,20]. However, most HCWs are neutral about their job commitment being affected by heat stress. Thermal discomfort may therefore negatively impact their roles as HCWs, which require considerable amounts of attention and commitment. Combined with a rising patient count, HCWs who continue working will not only worsen heat stress effects on themselves but also adversely affect the patients under their care due to poorer judgment. This is especially worrying during a pandemic setting as the burden of disease places an increased pressure on healthcare systems and personnel that would be further compounded by poor judgment of thermally strained HCWs when dealing with increased patient loads.

Wearing PPE for long durations in hot and humid environments can likely induce additional thermal strain. We observed HCWs frequently reported feelings of thirst, excessive sweating, exhaustion, and wanting to go to the comfort zone during their shifts. Other symptoms reported were headaches, dizziness, breathing difficulties, and dehydration, which are symptoms of heat-related illnesses. Other studies have demonstrated that increased thermal strain precipitates into heat injury [21] and causes exhaustion and fatigue [22]. Moreover, HCWs may not consider the symptoms they experienced as precursors to heat-related illnesses. Our results reveal an under-reporting of sick leave, which may be due to misunderstanding heat injury symptoms. Instead, HCWs may attribute symptoms experienced to other causes like increased cardiovascular stress. Subsequently, HCWs who continue to work despite expressing thermal strain symptoms are at an even greater risk of heat-related illnesses.

Overall, more respondents in India reported symptoms of heat-related illness than in Singapore. Some possible reasons include higher median WBGT reading observed in India and higher prevalence of dedicated rest areas, PPE removal during breaks, and air-conditioned worksites in Singapore. It is plausible that developed countries, like Singapore, have better infrastructure and systems to mitigate heat stress. On the contrary, developing countries like India tend to be under-resourced and thus underprepared to manage the effects of heat stress.

Current heat mitigation strategies include ensuring proper work–rest cycle and PPE removal during breaks. While acknowledging that work–rest balance may improve heat tolerance, HCWs indicated that they avoid taking breaks due to busy schedules, PPE conservation, or infection control. This may be exacerbated in countries where PPE is not as readily available and PPE conservation is more important. A recent study that surveyed HCWs working on COVID-19 worldwide found that more than half of HCWs reported shortages of at least one form of PPE and 30% reported that PPE was reused or washed due to shortages [23]. Moreover, HCWs may prefer not to doff PPE during breaks due to long removal durations and potential infection risks [24]. Therefore, strategies aimed at ensuring work–rest balance or PPE removal might be unfeasible during a pandemic.

Another heat mitigation strategy is pre-cooling, which aims to reduce baseline core temperature (T_c_) before activity to extend heat storage capacity and delay the onset of fatigue. Pre-cooling can be achieved via ice slurry ingestion [25]. Thermal comfort rating improvements after ice slurry ingestion from our study is consistent with prior research showing that pre-cooling with ice slurry could alleviate thermal strain in individuals wearing PPE while working in hot and humid environments [26]. Moreover, the dual role of ice slurry to cool and hydrate HCWs renders it more beneficial than hydration with fluids. This may contribute to extended work tolerance times for HCWs wearing PPE during a pandemic. In countries where ice slurry might not be available, cold water may be used for pre-cooling instead [27].

Other heat alleviation strategies include improving aerobic fitness and hydration. Although HCWs from this study agreed that both hydration and aerobic fitness would increase heat tolerance, more perceived hydration as a better strategy than keeping fit. This contrasts with findings from a recent meta-analysis which showed that the most effective heat mitigation strategy was improving aerobic fitness, with hydration being least effective [28]. Improving aerobic fitness can lower T_c_ [29], enhance heat dissipation mechanisms [30], and increase tolerance of a greater end T_c_ without any adverse pathophysiological effects [31]. These physiological benefits could translate into improved work tolerance time for HCWs spending long hours conducting TCAs in PPE. Moving forward, it may behoove employers of HCWs to take ownership of employee well-being and aerobic fitness to optimize HCWs’ heat tolerance [32].

It will also be essential to integrate the national heat-health action plans, specifically the heat-health warning systems (HHWSs), while considering current measures to reduce COVID-19 transmission. This is already used in many countries to alert the population most vulnerable to heat stress. As reported in a recent study [33], an adaptation strategy might involve a specific HHWS with personalized information based on individual (e.g., weight, height, age), behavioral (type of activity carried out and clothing worn), and environmental (work location) characteristics [34]. Internationally, projects that aim to alleviate the heat stress situation by customizing the forecast of heat risks and providing useful advice include the ongoing European HEAT-SHIELD project (https://www.heat-shield.eu/) and the Italian WORKLIMATE project (https://www.worklimate.it/). Hence, efforts in this study can be further expanded using the WORKLIMATE project.

However, the present study might have limitations. Selection bias might have occurred due to nonrandom sampling during data collection. Additionally, HCWs were required to self-report symptoms experienced. A proportion of HCWs who participated in the questionnaire held operational and sanitary roles. As these individuals were not medical professionals, the accuracy of self-reported symptoms might be affected. Moreover, the study was a real-time pandemic profiling of HCWs which posed various constraints that contribute to the study limitations. Firstly, the heavy workload of HCWs battling the pandemic might have prevented HCWs from participating in the study, resulting in a disparity in participant numbers between Singapore and India. Secondly, the ability to expand the use of ice slurry ingestion to HCWs working in India was limited as the study could not dictate the type of heat mitigation strategies that were employed at the respective hospitals in India and Singapore during pandemic conditions. Finally, the study was unable to provide a control group comparison of HCWs without PPE as PPE usage was compulsory for HCWs during this pandemic.

## 5. Conclusions

HCWs are aware of the potential dangers of working under hot and humid conditions while wearing PPE; they are also aware of how to manage these adverse effects and improve heat tolerance. However, alternative heat mitigation strategies may be necessary as current practices might be impractical. The risk of heat-related illness may be worsened in developing countries, where resources to combat heat stress are less readily available. Future studies should delineate whether adverse effects of heat stress on productivity and judgment of HCWs can impact patient safety and care.

## Figures and Tables

**Table 1:** Comparison of demographic details, roles, and location of workplaces among healthcare worker (HCW) respondents during the coronavirus disease 2019 (COVID-19) pandemic. (W = Wilcoxon rank-sum test statistics, χ^2^ = Pearson chi-square test statistics).

Factors	India	Singapore
	*n* = 110	*n* = 55
Age (years)	31.0 (26.0–38.0)	29.0 (27.0–33.0)
W = 3075.5, *p* = 0.48		
Male gender	64 (58%)	20 (36.4%) *
χ^2^(1) = 10.9, *p* < 0.01		
BMI (kg/m^2^)	24.4 (22.5–26.6)	22.0 (18.8–24.8) *
W = 3569, *p* = 0.014		
WBGT (°C)	30.2 (29.1–31.8)	20.0 (19.3–26.05) ***
W = 5589.5, *p* < 0.01		
Role		
Medical	45 (39%)	21 (42%)
Nursing	33 (29%)	28 (56%)
Operations	4 (4%)	0 (0%)
Sanitary	5 (4%)	0 (0%)
Others	28 (24%)	1 (2%)
Location		
Tentage	2 (2%)	26 (52%)
Fever Facility (FF)	24 (21%)	26 (52%)
Clean Area	25 (22%)	14 (28%)
Others	64 (57%)	2 (4%)

* *p* < 0.05; *** *p* < 0.001, when compared with India. Data expressed in *n* (%) for categorical variables, mean (standard deviation (SD)) and median (interquartile range (IQR)) for continuous variables.

**Table 2:** Comparison of impact of PPE usage and related thermal stress, duration of usage, time taken for donning and doffing the PPE, whether or not PPE could be removed during breaks, availability of dedicated rest areas, and symptoms experienced due to heat stress (W = Wilcoxon rank-sum test statistics, χ^2^ = Pearson’s chi-square test statistics).

Factors	India	Singapore
	*n* = 110	*n* = 55
Days/week in PPE	6 (5–6)	5 (4–5) ***
W = 3854, *p <* 0.01		
Hours/shift in PPE	6 (5–8)	8 (8–8.15) ***
W = 1516, *p <* 0.01		
Working in A/C	27 (25%)	34 (62%) ***
χ^2^(1) = 20.29, *p* < 0.01		
Dedicated rest area	46 (42%)	55 (100%) ***
χ^2^(1) = 53.91, *p* < 0.01		
Time taken to don PPE (minutes)	7 (5–10)	3 (2–5) ***
W = 4850.5, *p* < 0.01		
Remove PPE on breaks	66 (60%)	54 (98.2%) ***
χ^2^(1) = 25.06, *p* < 0.01		
Symptoms in PPE		
Headache	36 (33%)	12 (22%)
χ^2^(1) = 1.62, *p* = 0.20		
Dizziness	7 (6%)	16 (29%) ***
χ^2^(1) = 13.95, *p* < 0.01		
Thirst	98 (85%)	46 (92%)
χ^2^(1) = 0.8973, *p* = 0.34		
Vomiting	0 (0%)	0 (0%)
Excessive sweating	95 (86%)	50 (90%)
χ^2^(1) = 0.3485, *p* = 0.56		
Breathing difficulty	27 (25%)	7 (13%)
χ^2^(1) = 2.45, *p* = 0.12		
Dehydration	27 (23%)	3 (6%) **
χ^2^(1) = 7.75, *p* < 0.01		
Exhaustion	86 (78%)	42 (76%)
χ^2^(1) = 0.452, *p* = 0.76		
Wanting to go comfort zone	94 (86%)	42 (76%)
χ^2^(1) = 1.51, *p* = 0.22		
Sick leave	3 (3%)	4 (8%)
Fisher’s exact: *p* = 0.22		

** *p* < 0.01; *** *p* < 0.001, when compared with India. Data expressed in *n* (%) for categorical variables and median (IQR) for continuous variables.
